# The Diverse Applications of Cladistic Analysis of Molecular Evolution, with Special Reference to Nested Clade Analysis

**DOI:** 10.3390/ijms11010124

**Published:** 2010-01-08

**Authors:** Alan R. Templeton

**Affiliations:** Department of Biology, Washington University, St. Louis, MO 63130-4899, USA; E-Mail: temple_a@wustl.edu; Tel.: +1-314-935-6868; Fax: +1-314-935-4432

**Keywords:** association studies, phylogeography, human evolution, conservation biology, species

## Abstract

The genetic variation found in small regions of the genomes of many species can be arranged into haplotype trees that reflect the evolutionary genealogy of the DNA lineages found in that region and the accumulation of mutations on those lineages. This review demonstrates some of the many ways in which clades (branches) of haplotype trees have been applied in recent years, including the study of genotype/phenotype associations at candidate loci and in genome-wide association studies, the phylogeographic history of species, human evolution, the conservation of endangered species, and the identification of species.

## 1. Introduction

Clade comes from the Greek word for “branch”. In evolutionary biology, clade refers to a branch of an evolutionary tree. Cladistics refers to analytical techniques that make use of clades in evolutionary trees. One of the first uses of the word cladistics in evolutionary biology was to an analytical method of estimating the evolutionary tree itself through shared, derived characters that would define clades [1]. In this review, the focus is not on tree estimation itself (see [2] for a comparison of several tree estimation algorithms), but rather on using the clades of an already estimated tree in the analysis of other types of data.

Traditionally, evolutionary trees have referred to trees of species. With the advent of molecular data, increasingly evolutionary trees are estimated for specific genes or regions of a genome both within and among species. This review is limited to evolutionary trees of genes or genomic regions. These DNA regions may be sampled within and/or between species, and both types of sampling will be considered in this review, but the emphasis will be upon intraspecific samples. Given a sample of homologous DNA sequences, the very definition of homology means that all the DNA sequences in the sample are descendants from a common ancestral DNA molecule. As one traces the current sample of DNA sequences into the past, DNA lineages coalesce, reflecting descent from a common ancestral molecule. With each coalescent event, the number of DNA lineages is reduced by one until ultimately all the current DNA lineages coalesce to a single common ancestral molecule. This detailed genealogy of a sample of DNA sequences that is defined by a series of coalescent events is generally not observable [3]. What is observable is whether or not two copies in the current sample of DNA sequences differ or not. If they differ, one or more mutations had occurred in one or both of the DNA lineages before they coalesced into a common ancestral molecule. If all the DNA replication events not marked by a mutational change in the DNA genealogy are collapsed together, a lower resolution version of the DNA genealogy emerges called a haplotype tree. In a haplotype tree each current DNA sequence and each node in the tree represents a distinct and unique sequence state called a haplotype. Every branch in this evolutionary tree of haplotypes is marked by one or more mutational events. Hence, the haplotype tree is a map that shows how all the current array of genetic variation found in the diverse haplotypes in the sample arose by the accumulation of mutations in DNA lineages over evolutionary history. This history can be obscured if recombination occurs, so haplotype trees are restricted to areas of the genome showing little to no recombination. This review focuses upon how clades of haplotypes in haplotype trees have been used to analyze a variety of data types in biology.

## 2. Genotype/Phenotype Associations

### 2.1. Basic Rationale for a Cladistic Approach

Suppose a sample of DNA sequences is obtained within a population from a single species. Suppose further that the DNA is sequenced from a region of the genome that has functional significance and that can influence or affect a trait of interest. To test if the current genetic variation found in this DNA region is associated with phenotypic variation in the trait of interest, one could look at all the individual nucleotide and indel variants and separately test each one for associations with the phenotypic variation. However, if this is a region of low to no recombination, the individual nucleotide polymorphisms are expected to show high magnitudes of linkage disequilibrium, a population-level correlation between different polymorphic sites. Linkage disequilibrium arises automatically when a new haplotype is created through mutation. When a mutation first occurs, it is on one and only sequence background and hence shows much linkage disequilibrium with pre-existing polymorphic sites. With no subsequent recombination, that initial association between the mutation and the pre-existing genetic variants that were on its chromosome of origin will never break down. Hence, the individual polymorphic sites are not statistically independent, which complicates tests for phenotypic association. Another difficulty caused by linkage disequilibrium is encountered in trying to go from association to causation. Typically not all variation in the DNA region of interest is scored and/or variation may exist in adjacent DNA regions that were not sequenced but that still show linkage disequilibrium with variants within the sequenced region. This means that a scored variant that displays a strong phenotypic association cannot be assumed to be the causative mutation; it may be causative but it may simply be in linkage disequilibrium with an unscored variant [4]. Moreover, when a specific scored mutation does show a significant phenotypic association, it does not signify that the causative mutation is close-by in the genome. In areas of low to no recombination, there is little correlation between physical proximity in the DNA molecule with the magnitude of linkage disequilibrium. Instead, the magnitude of disequilibrium is more reflective of the proximity of mutations in the haplotype tree (*i.e.*, time) rather than proximity in the genome (space). As a consequence, single polymorphic site analyses can be actively misleading in areas of low to no recombination [3].

Another alternative is to analyze the haplotypes themselves for phenotypic associations. A haplotype is defined by the simultaneous state of all polymorphic sites within the sequenced region, so linkage disequilibrium is implicitly taken into account. However, the number of haplotypes found within a DNA region is often large. Suppose there are *n* haplotypes in the sample. Then, there are ½*n*(*n–* 1) comparisons between haplotypes to examine for differences in phenotypic associations. Note that the number of haplotype comparisons increases proportionally to *n^2^*. Such a large number of comparisons quickly erodes statistical power.

**Figure 1.**
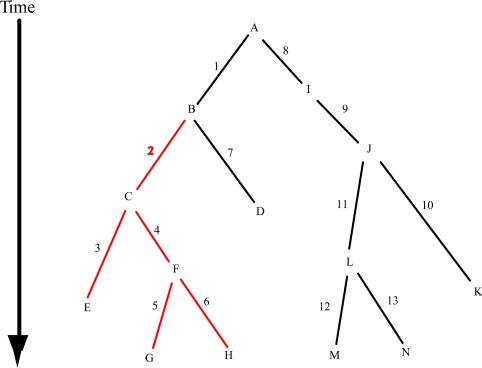
A hypothetical haplotype tree. Haplotypes are indicated by letters, and the mutations that generated the various haplotypes from the ancestral type A are indicated by numbered branches. Mutation numbers shown in black are neutral with respect to the phenotype of interest, but mutation 2, shown in red and producing haplotype C, is assumed to be associated with a phenotypic change. This phenotypically important mutation is shared by all the other haplotypes that descend from haplotype C, as shown by the clade of haplotypes defined by red branches.

Haplotype trees can greatly reduce the dimensionality of this problem. Under the comparative method of evolutionary biology, the most relevant contrasts are between adjacent nodes in an evolutionary tree. A fully resolved evolutionary tree of *n* haplotypes has *n-*1 branches connecting the haplotypes, so at most there are only *n*– 1 evolutionarily meaningful contrasts, although ambiguities in tree estimation and/or limited recombination may increase this number somewhat [5]. The rationale for this is shown in Figure 1. Most mutations probably have no functional or phenotypic significance, but occasionally functionally important mutations occurred in evolution. In the absence of recombination and back-mutation, a functionally significant mutation is shared by all the haplotypes in the clade that is defined by the branch in the haplotype tree upon which the original, functionally important mutation occurred, as shown in Figure 1. The fundamental premise of cladistic analysis of genotype/phenotype associations is that *evolutionarily closely related haplotypes will tend to share phenotypically important mutations*. Hence, tests for phenotypic association should be limited to contrasts separated by a branch in the haplotype tree. It is a waste of statistical power to contrast, for example, haplotype E with haplotype K in Figure 1. There are only *n*-1 contrasts across branches in a haplotype tree, which represents a tremendous reduction in dimensionality from ½*n*(*n–* 1) when the number of haplotypes is large, as in now common with high resolution genetic surveying techniques. This reduction in dimensionality alone greatly augments the statistical power of a cladistic approach by avoiding the squandering of statistical power on evolutionarily uninformative contrasts. Moreover, although it is not possible to localize the causative mutation in genomic space, a cladistic analysis can localize the association in evolutionary time and identify the haplotype contrast that is most likely to differ by the smallest number of mutations in addition to the causative mutation (haploytpes B versus C in Figure 1). This in turn can greatly aid in identifying the causative mutation [6,7]. Finally, sometimes independent mutations converge to the same phenotypic effect. A cladistic analysis can identify these convergent mutations whereas they are invisible to analyses that do not utilize information about evolutionary history [8].

### 2.2. Nested Clade Analysis

To take advantage of these many optimal properties of a cladistic analysis of genotype/phenotype associations it is necessary to have a statistical method that covers the *n*-1 evolutionarily relevant statistical contrasts. The first statistical procedure used for this purpose was nested clade analysis [8]. This procedure converts the haplotype tree into a fully nested statistical design by taking advantage of the fact that a tree has branches upon larger branches upon even larger branches, *etc*. Starting at the tips of a haplotype tree, the nesting is initiated by moving one mutational step into the interior of the tree and taking the union of all haplotypes that share a common node by stepping back by this one mutational step. These unions are called one-step clades. These one-step clades are then pruned off, leaving only the more interior parts of the haplotype tree (if any haplotypes remain unnested). The nesting procedure is then executed on the pruned tree to create interior one step clades (if needed), and these pruning and nesting algorithms are repeated until all haplotypes are nested. Sometimes a haplotype may be stranded by this procedure, and special nesting rules are used in this case and also to accommodate ambiguities in the estimated haplotype tree [9]. In the hypothetical tree shown in Figure 1, the one step clades created by moving in one mutational step from the tips are one-step clade 1–1 (haplotypes F, G, H); 1–2 (C, E); 1–3 (B, D); 1–4 (L, M, N); and 1–5 (J, K). Pruning off these one-step clades, the only remaining part of the tree is A–I, so haplotypes A and I are then nested together to form one-step clade 1–6 (A, I). The first level of contrasts in the nested clade analysis is to contrast the adjacent haplotypes *within* each one-step clade; e.g., contrast G *vs.* F, and H *vs.* F in 1-1; contrast E *vs*. C in 1–2; *etc*. Note that this first level of nested clade analysis makes 8 contrasts involving the 14 haplotypes in the tree.

**Figure 2.**
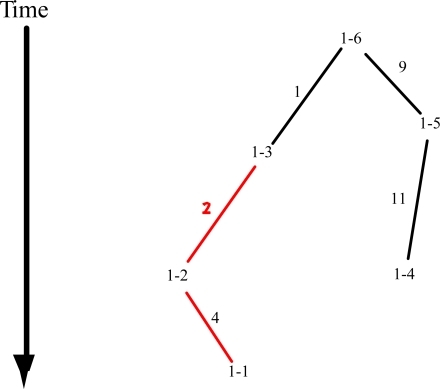
A hypothetical evolution tree of one-step clades derived from the haplotype tree shown in Figure 1.

The next level in a nested clade analysis is to regard each one-step clade as the genetic units and not individual haplotypes. Figure 2 shows the evolutionary tree of one-step clades derived from the haplotype tree shown in Figure 1. The same nesting algorithm applied to haplotypes is now applied to one-step clades to produce a series of two step clades: 2–1 (1–1, 1–2); 2–2 (1–4, 1–5); and 2–3 (1–3, 1–6). The second level of analysis is to contrast the adjacent one-step clades nested within the same two-step clade. There are three contrasts at this level. If a tree of two-step clades is then constructed, the nesting algorithm would pool them into a single three-step clade. Hence, the third level of analysis is to contrast the adjacent two-step clades nested within a three-step clade, which in this example results in two contrasts: 2–1 *vs.* 2–3; and 2–2 *vs.* 2–3. Note that the total number of contrasts is 13, which is the number of haplotypes minus one. Hence, the nested analysis makes all *n*-1 evolutionarily informative contrasts. In this hypothetical example, the contrast that would detect the phenotype effect is the contrast of 2–1 *vs.* 2–3 because clade 2–1 corresponds to the red-clade in Figure 1. The cladistic analysis would also identify branch 2 as the branch associated with the phenotypic transition. The major advantage of the nested clade approach is that the nested contrasts are all independent under the null hypothesis of no phenotypic associations [10,11], making it easy to correct for multiple testing through a Bonferroni-Zidak correction. The exact nature of the statistical tests used in the cladistic contrasts varies with the data type, sampling design, and the nature of the genotypes. For example, a standard nested analysis of variance is used for quantitative phenotypes in homozygous or haploid strains [8,12], a permutational analysis is used for quantitative phenotypes measured on diploid individuals with much heterozygosity [13]; contingency tests are used for categorical and case/control data [10,14,15]; and likelihoods or transmission-disequilibrium analyses are used for family/pedigree data [16,17].

### 2.3. Other Evolutionary Methods for Genotype/Phenotype Associations

Another simple method for making the evolutionarily relevant contrasts is tree-scanning. In tree-scanning, a single branch in the haplotype tree is cut to divide the haplotype tree into two pieces, each of which is then treated as an “allele” in the equivalent of a single-locus gentoype/phenotype association test [5]. For example, cutting branch 2 in the tree shown in Figure 1 would result in treating the red colored clade of haplotypes as an allele and all the haplotypes in the black colored portion of the haplotype tree as the second allele. This contrast should yield a strong phenotypic association. In contrast, cutting branch 1 would pool the red-colored class with some of the black portion of the tree into a single allele, and thus result in a weaker phenotypic signal. Tree-scanning is easy to implement with a wide variety of data types and sampling designs, but unlike the nested clade approach, the contrasts are not independent. Consequently, multiple corrections for correlated contrasts are made with procedures that make use of extensive permutation testing [5].

A wide variety of other methods for making evolutionarily relevant contrasts have been proposed since the original nested clade procedure was published [18–42]. These studies when coupled with the results of nested clade analyses clearly document the increased power and ability to detect and localize genetic variants of phenotypic importance. Because many of these cladistic procedures can now be applied at the level of whole genome scans and with the discovery that much of the human genome is contained in haplotype blocks of little to no recombination [43], the applicability of cladistic approaches to genotype/phenotype association studies is increasing dramatically.

## 3. Phylogeography and Associated Applications

### 3.1. Nested Clade Phylogeographic Analysis

Nested clade analysis provides a highly flexible method for testing associations between a haplotype tree and other types of data. Although nested clade analysis was developed and initially used for phenotypic associations, there is no reason to limit this approach just to individual phenotypic data. Another type of data is simply the geographical location(s) where a haplotype is found, and in this case nested clade analysis can be used to test the null hypothesis that there is no association between the haplotype tree and geography. Geographical associations with haplotypes can arise for two reasons. First, geographical associations arise from a species’ demographic structure and history. When a mutation first occurs to create a new haplotype, that haplotype is obviously restricted to its geographical site of origin. However, once a new haplotype exists and is replicated, it can spread through space and time, and the dynamics of this spread depend upon the amount and pattern of gene flow within the species and historical factors such as fragmentation that would prevent a haplotype originating in one region from spreading into another or range expansion that could place the haplotype into a new geographical area. Geographical associations arising from a species’ demographic structure and history are not expected to be locus specific, as these demographic and historical factors should affect all loci. The second cause for geographical association is locus-specific. If natural selection is occurring on a haplotype or haplotype clade at a locus, selection can influence its spatial distribution, either by accelerating its spread throughout the species or by restricting the selected haplotype to certain areas where it is locally adaptive. Intraspecific phylogeography focuses upon a species’ historical demography and events that influence how genetic variation is distributed in space and time. Therefore, the haplotype tree/geography associations that are general and not locus specific are the ones that are informative about a species’ phylogeography.

Nested clade analysis was modified to test the null hypothesis of no association between the clades in a haplotype tree with geography [44]. When the null hypothesis of no association is rejected, the observed patterns of statistical significance are biologically interpreted using explicit, *a priori* criteria derived from sampling and coalescent theory. These predictions have been validated through the use of 150 positive controls, making nested clade phylogeographic analysis the most extensively validated method for phylogeographic inference [45]. Originally, nested clade phylogeographic analysis was only applied to single DNA regions (typically mitochondrial DNA), so there was no way to check if the patterns observed were general or locus specific. This problem was eliminated by the development of multi-locus nested clade phylogeographic analysis that eliminates locus specific patterns through a cross-validation procedure [46]. The cross-validation procedure is also effective at eliminating false positives [46,47]. Moreover, a likelihood framework based on coalescent theory was developed for the multi-locus version of nested clade phylogeographic analysis that allows every cross-validated interpretation to be explicitly tested as a null hypothesis using log-likelihood ratio tests [48,49]. This log-likelihood ratio testing-framework is highly flexible and also allows the testing of *a priori* phylogeographic hypotheses.

One of the most unique features of nested clade phylogeographic analysis is that it uses the coalescent information contained in the genetic data to infer and test phylogeographic events and processes. Haplotype trees are the units of analysis, and such trees represent the estimable portion of the coalescent process at a given DNA region. In contrast, most other phylogeographic techniques make no use of the historical information contained in the genetic data but rather merely look at goodness of fit of phylogeographic scenarios to genetic measures such as heterozygosity, number of alleles, *etc.*, that contain little to no historical information [50]. As a result, these methods have to provide the historical framework *a priori* by specifying highly detailed phylogeographic scenarios using multiple parameters, most of which are unknown and indeed unknowable [51]. Because nested clade phylogeographic analysis requires no *a priori* models, it can uniquely discover new or unanticipated events in a species’ evolutionary history.

### 3.2. Nested Clade Phylogeographic Analysis of Human Evolution

**Figure 3.**
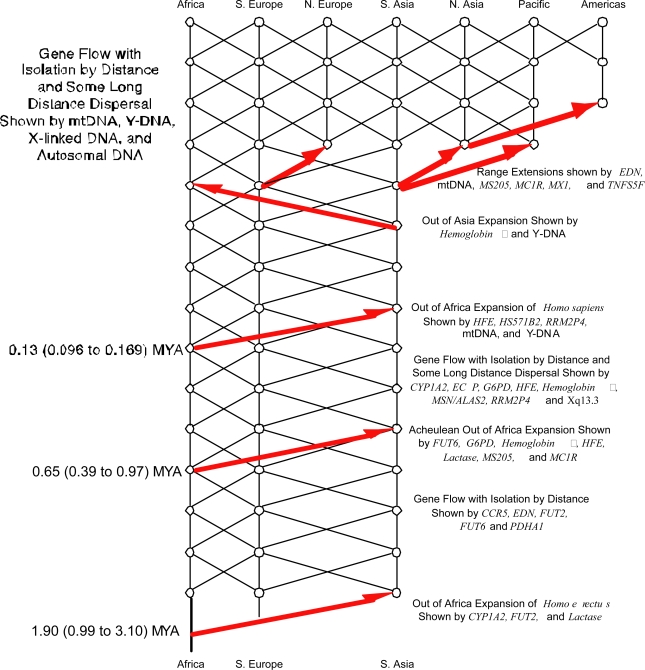
The cross-validated, statistically significant inferences in human evolution over the past 2 million years from nested clade phylogeographic analysis [52,54]. Vertical black lines indicate genetic descent in a location, whereas diagonal black lines indicate gene flow between different areas. Red arrows indicate significant range or population expansions.

The ability of nested clade analysis to discover unanticipated phylogeographic events is demonstrated by an analysis of 25 DNA regions to infer human phylogeography [52–54]. The results of this analysis are summarized in Figure 3. The nested clade analysis discovered features of human evolution that had not been proposed in any of the common models of human evolution that have dominated the literature over the last few decades. One such novel feature was a population expansion out of Africa into Eurasia that was dated molecularly to 650,000 years ago. However, once proposed, this expansion is concordant with the archaeological data of the sudden expansion of the Acheulean culture out of Africa and into Eurasia during a climatically favorable window in time [53]. The nested clade analysis also allowed many *a priori* hypotheses about human evolution to be statistically tested that had never before been subject to testing as a null hypothesis. For example, one of the dominant views of human evolution is that the latest expansion out-of-Africa (the one dated to 130,000 years ago in Figure 3) was characterized by the expanding African population driving to complete genetic extinction all of the Eurasian populations (the “replacement” hypothesis). Nested clade analysis allowed the first (and only) testing of the out-of-Africa replacement model as a null hypothesis, and replacement was strongly rejected with a p-level of less than 10^−17^ [52,55]. Hence, there was some degree of interbreeding, not total replacement, between the expanding African population with the Eurasian populations.

### 3.3. Nested Clade Phylogeographic Analysis in Conservation Biology

Another use of nested clade phylogeographic analysis is in the area of conservation biology. One application is to identify Evolutionary Significant Units (ESU) [56], an important management unit in conservation biology. For example, African elephants are formally subdivided into two subspecies, the savanna and forest forms. To be considered different ESUs, it is necessary to show that these two groups of African elephants are genetically differentiated from one another and have been behaving effectively as separate evolutionary lineages. However, it is known that bull savanna elephants can and do mate with forest females, and the fertile, female offspring of such matings can become incorporated into the breeding groups of savanna elephants [57]. Hence, the two groups are not absolutely isolated reproductively, but it is still possible that gene flow and introgression have been reduced to such low levels that the two groups are effectively evolving as separate lineages. Nested clade phylogeographic analysis is ideal for testing for ESUs because it uses statistical criteria to detect fragmentation (long-term genetic isolation to a sufficient degree to define different lineages) that allows for the possibility of a small degree of genetic introgression. Also, in recent lineages, lineage sorting during the coalescent process is commonplace that can cause the haplotype tree to not correspond topologically to the lineage tree [3]. Because nested clade analysis is based on local, nested contrasts within the haplotype tree and not the overall tree topology, its inferences are robust to lineage sorting. Nested clade analysis was applied to five DNA regions sampled from African elephant populations throughout Africa [57], and all five DNA regions lead to the inference of significant fragmentation between forest and savanna elephants [51]. The null hypothesis that all five inferences were indeed due to a single fragmentation could not be rejected with a log-likelihood ratio test despite the fact that all five DNA regions had haplotype trees with different topologies for the forest and savanna taxa, thereby illustrating that nested clade analysis is indeed an excellent tool for inferring lineages even in the face of limited introgression and lineage sorting. Hence, the forest and savanna forms of African elephants are distinct ESUs and need to be managed as separate entities.

Another application of nested clade phylogeographic analysis to conservation is to infer how environmental factors in a landscape and the ecological attributes of the species living in this landscape affect the population structures of the species. For example, nested clade phylogeographic analysis was performed on several bovid species and elephants, all inhabiting the same general area in Eastern Africa [58]. Some species displayed a pattern of isolation-by-distance over this landscape, whereas others displayed significant fragmentation with strong barriers to gene flow. Studies on the dispersal behavior of current populations were not informative about these differences. However, the feeding ecology of the species was highly predictive of the observed phylogeographic patterns, with feeding generalists displaying the isolation-by-distance pattern and feeding specialists (specifically to savanna plants) the fragmented pattern. Moreover, the areas that behaved as strong barriers to gene flow corresponded to areas in which the preferred food plants of the feeding specialists were absent. Thus, differences in feeding ecology and habitat patchiness explained well the differing genetic patterns observed in these species living in the same landscape. These conclusions had many conservation implications for the management of these bovid species. For example, species showing long-term fragmentation are more likely to have subspecies and local adaptation, so translocation of animals between fragmented areas should be avoided or undertaken cautiously, whereas this restriction is not applicable to species showing genetic continuity over this landscape. It was also concluded that the savanna specialists do not occupy all appropriate habitat patches at any given time because of their inability to disperse across non-savanna habitat. Hence, these unoccupied savanna areas are appropriate sites for establishing new populations of endangered, savanna specialist species. By elucidating processes and not mere patterns, nested clade phylogeographic analysis allowed insight into how ecosystems function over evolutionary time and the ability to make better management recommendations for the ecosystem as a whole and for the endangered species within it.

## 4. Species Identification

Species are the fundamental units of much of biology, yet there is still no consensus on the definition of a species nor how to identify them. Ideally, a species concept should be related to evolutionary theory (rather than just an arbitrary taxonomic convenience), be general, and be applicable in a practical manner [59]. The only species concept that satisfies all three of these criteria is the cohesion species concept [60,61]. A cohesion species is an evolutionary lineage that maintains its cohesiveness over time because it is a reproductive community capable of exchanging gametes and/or an ecological community sharing a derived adaptation or adaptations needed for successful reproduction. The cohesion species is defined in terms of evolutionary lineages, and thus is related to a fundamental aspect of evolutionary theory. Since all life, both sexual and asexual, forms lineages, the cohesion concept is general. In terms of practical applicability, the cohesion concept can be applied in a scientifically rigorous fashion by rephrasing it as a set of testable null hypotheses. These hypotheses are: (1) the organisms sampled are derived from a single evolutionary lineage; and (2) if more than one lineage is identified by rejecting hypothesis 1, then the identified lineages are a single reproductive community and/or a single ecological community. Both of these null hypotheses can be tested with cladistic analyses.

As already shown in Section 3.3 with the elephant example, nested clade phylogeographic analysis can test for past fragmentation. Fragmentation means that the organisms sampled are subdivided into two or more evolutionary lineages, so whenever fragmentation is inferred in a statistically significant fashion, the first null hypothesis that the sampled organisms are derived from a single evolutionary lineage is rejected. As also illustrated by the elephant example, the inference of fragmentation does not exclude the possibility of some limited gene flow or introgression among the lineages, nor does it require that the haplotype tree correspond to a tree of lineages. These are optimal properties when trying to infer the species status of recently fragmented groups. Indeed, not all haplotype trees correspond to the species tree for humans, chimpanzees and gorillas [62], so demanding that haplotype trees correspond to the species tree is a biologically unrealistic requirement for species status.

Given that hypothesis 1 is rejected, the next step is to test whether or not the evolutionary lineages correspond to a single reproductive and/or ecological community. This phase of testing can also be done with cladistic analysis, but now using the same tools described in Section 2 for testing genotype/phenotype associations. In Section 2, the haplotype tree for a candidate locus was used to test for phenotypic associations related to the function of that locus in a sample of individuals from the same species and without population stratification, as stratification is a well-known source of artifacts in genotype/phenotype association testing [63]. Given that hypothesis 1 has been rejected, the sample in this case is highly stratified into distinguishable evolutionary lineages. These lineages may differ for reproductive and/or ecological traits. For the purpose of species identification, it is not necessary to have candidate loci for these traits; it is merely enough that these traits be associated with the lineages defined by testing hypothesis 1. Hence, the artifacts that arise in genotype/phenotype association studies due to stratification are now utilized in a positive fashion to see if there are significant associations of the lineages with important reproductive and/or ecological traits. Because these associations arise from stratification, the associations, if present, should be detectable with virtually any DNA region and not just candidate genes. The main problem is to identify traits of reproductive and/or ecological significance.

As an example, nested clade phylogeographic analysis of mitochondrial DNA in mole rats sampled throughout Israel indicated the presence of at least three statistically significant evolutionary lineages defined by two significant fragmentation events even though there was some introgression and/or lineage sorting of ancestral polymorphisms [64]. Focusing first on traits that can influence reproductive communities, one excellent candidate is chromosome number. Differences in chromosome number directly reduce the fertility of hybrids [65], and hence these differences act as a post-mating reproductive barrier. A nested clade analysis was performed on the phenotype of chromosome number. Three significant changes in chromosome number were detected, with two of them corresponding exactly to the two fragmentation events that define the three evolutionary lineages detected in testing hypothesis 1. The third chromosome number transition corresponds to a range expansion, and could indicate a speciation event in process. A second class of traits that can define reproductive communities are pre-mating isolating barriers. One such potential trait is the vocal courtship calls of male mole rats that are used in mate recognition. Both fragmentation events were associated with significant changes in male courtship songs. Hence, the three evolutionary lineages defined by the two fragmentation events inferred from nested clade phylogeographic analysis are also concordant with significant changes in both a post-mating isolating barrier (chromosome number) and a pre-mating isolating barrier (male courtship song). Ecological traits can be used to test whether or not these evolutionary lineages define ecological communities. The different mole rat lineages inhabit areas that differ in temperature and rainfall, and these physical variables directly and strongly influence the environment in which these fossorial mammals live. E. Nevo and his co-workers identified seven candidate traits that are of ecological significance [64]. Cladistic contrasts reveal that the three evolutionary lineages also differ significantly in one or more of these ecological traits. Hence, the second null hypothesis that the evolutionary lineages are a single reproductive community and/or single ecological community is rejected on both reproductive and ecological grounds. Hence, there are at least three cohesion species of mole rats in Israel.

This method of inferring species has many strengths. First, it is based upon hard scientific inference; that is, the falsification of hypotheses. Second, it makes all the data and the inferences based upon them completely explicit. There are no hidden assumptions or subjective inferences. Third, the very act of testing the two null hypotheses leads to much insight into the nature of the speciation event and its evolutionary consequences. Fourth, if the data are inadequate for falsifying either null hypothesis, the explicit nature of the cladistic analysis indicates what data would be most valuable to gather in future studies.

## 5. Conclusions

Biology is different from disciplines such as chemistry or physics because all living forms have a history, and that history has played a critical role in shaping the present. Historical effects can complicate many analyses, but these same histories can be used to augment analytical power. Cladistics is a method that uses evolutionary history to augment analytical power. The evolutionary histories of DNA regions are utilized by cladistic analyses to reduce dimensionality, concentrate statistical power, detect parallelisms that are invisible to non-historical analyses, and bring hard inference based upon falsification of hypotheses to disciplines that have rarely used hard inference in the past. As shown in the previous Sections, cladistic analytical methods can be applied to a broad array of problems in genetic epidemiology, conservation biology, basic evolutionary biology, and species inference. Similarly, a broad array of data types is amenable to cladistic analyses; including continuous, categorical, and spatial variables. Although cladistics is about how to use the past to understand the present, these optimal properties of cladistic methodologies coupled with the breadth of their applicability ensure that cladistic analyses will have a productive future as an important analytical tool in biology.
